# Phytochemistry, quality control and medicinal uses of Saffron (*Crocus sativus L*.): an updated review

**DOI:** 10.25122/jml-2022-0353

**Published:** 2023-06

**Authors:** Rabha Aissa, Mohamed Ibourki, Hasna Ait Bouzid, Laila Bijla, Samira Oubannin, El Hassan Sakar, Simohamed Jadouali, Andi Hermansyah, Khang Wen Goh, Long Chiau Ming, Abdelhakim Bouyahya, Said Gharby

**Affiliations:** 1 Department of Bio-Industrial Engineering & Environment, Bioprocesses and Environment Team, Superior School of Technology, Ibn Zohr University, Agadir, Morocco.; 2 Biotechnology, Analytical Sciences and Quality Control Team, Laboratory of Analysis Modeling, Engineering, Natural Substances and Environment, Polydisciplinary Faculty of Taroudant, University Ibn Zohr, Agadir, Morocco.; 3 Laboratory of Biology, Ecology, and Health, Faculty of Sciences, Abdelmalek Essaadi University, Tetouan, Morocco.; 4 Laboratory of Biotechnology, Bioanalysis and Bioinformatics, Superior School of Technology, Sultan Moulay Slimane University, Khenifra, Morocco.; 5 Department of Pharmacy Practice, Faculty of Pharmacy, Universitas Airlangga, Surabaya, Indonesia; 6 Faculty of Data Science and Information Technology, INTI International University, Nilai, Malaysia; 7 School of Medical and Life Sciences, Sunway University, Sunway City, Malaysia; 8 PAPRSB Institute of Health Sciences, Universiti Brunei Darussalam, Gadong, Brunei Darussalam; 9 Department of Biology, Faculty of Sciences, Mohammed V University, Rabat, Morocco

**Keywords:** adulteration, chemical composition, heart disease, saffron

## Abstract

Saffron, botanically known as *Crocus sativus L*., is renowned as the world’s most expensive spice and has been utilized in various fields since ancient times. Extensive scientific research has been conducted on *Crocus sativus (C. sativus)*, focusing on its phytochemical composition, diverse applications, and biological activities. *C. sativus* phytochemicals consist mainly of three compounds, namely crocin, picrocrocin, and safranal, which are responsible for most of its properties. Saffron is rich in bioactive compounds, more than 150 of which have been isolated. Owing to its unique composition and properties, saffron is used in various fields, such as the food industry, perfumery, cosmetics, pharmaceutics, and medicine. However, the high economic value of saffron makes it susceptible to adulteration and various fraudulent practices. To deal with this issue, a number of methods and techniques have been developed to authenticate and determine adulterants in saffron. This paper presents a bibliometric study of saffron based on the Web of Science database, analyzing 3,735 studies published between 2000 and 2021. The study also examined author participation and collaboration networks among countries. Production, transformation, chemical composition, methods of adulteration detection, uses, and health properties of saffron are also discussed.

## INTRODUCTION

Saffron (*Crocus sativus L*.) is a monocotyledonous herbaceous triploid plant with a chromosome count of 3n=24 (the basic chromosome number is 8). It belongs to the Iridaceae family and constitutes one of the 85 species of the *Crocus* genus [1]. *Crocus sativus L. (C. Sativus)* is known to be the world's most expensive spice. It has been cultivated for a long time in several countries in Asia, the Mediterranean basin, Europe, and South Africa [2]. Iran holds the leading position in saffron production and exportation, with 404 tons in 2018 [3]. The highly valued part of the plant is the stigma of its flowers [4], which is grown in greenhouses and fields of different soils. Saffron grows from a corm, which is considered the nutrient reserve of the plant [4].

From a chemical standpoint, saffron contains more than 150 chemical compounds, including flavonoids, carotenoids, flavonoid glycosides, monoterpenes and monoterpenoid derivatives, monocyclic aromatic hydrocarbons, amino acids, and alkaloids [5]. This remarkable composition contributed to its broad spectrum of medicinal effects [5–7]. Its use can be traced back to ancient civilizations such as the Egyptians and traditional Chinese medicine [8,9]. The precise chemical constitution of saffron and its health-promoting characteristics, such as antioxidant, hypolipidemic, antihypertensive, immunomodulatory, antimicrobial, antitumor, and antidepressant properties, have been determined and recently reviewed [10,11].

In addition to its medicinal, soporific, and culinary benefits, it is an important source of food dye as well as a perfume ingredient. Nowadays, saffron is used in many fields, including in the food industry (as a flavoring and coloring agent), in perfume compositions, medicine, healthcare, and cosmetics [7]. Due to its exceptional composition, extensive use, and the difficulties linked to its production, saffron is the most expensive spice on the world market. Approximately 60,000 flowers are required to produce 1 kg of dried stigma, estimated at 10,000$ USD [8]. This high price makes this product a target for various types of adulterations. To address this issue, different fraud detection methods have been developed [12]. While several reviews have been published on various aspects of saffron, none have comprehensively compiled its production, chemical composition, transformation, biological activities, health-healing properties, and quality assessment. This review aimed to fill that gap by providing a bibliometric analysis of the existing literature, highlighting saffron production, chemical composition, and biological activities, and focusing on strategies and emerging methods for ensuring its authenticity and quality control. In addition, we discussed the different uses of saffron.

## MATERIAL AND METHODS

The present study utilized the Web of Science (WoS) database as the primary data source, given its extensive collection of scholarly papers (n=3735). The investigation aimed to analyze scientific research studies published between 2000 and 2021, employing a bibliometric approach. Various aspects of the research were examined, including the global publication trend, identification of journals with the highest number of publications, assessment of prolific authors, evaluation of the most productive countries, and analysis of commonly used keywords. To conduct this study, the search string used was: (TS = ("saffron") OR TS = ("saffron") OR TS = ("Crocus sativus*")) AND PY = (2000-2021). VOS viewer and Origin Pro (version 2021) software were used to process bibliographic data.

## RESULTS

### Global publication trend (2000–2021)

**Figure 1:**
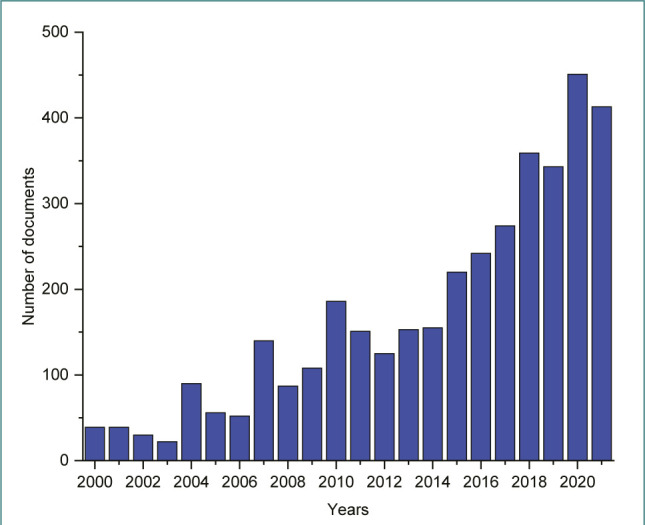
Global publication trend on *C. sativus* (2000-2021)

The analysis of the WoS database yielded a total of 3,735 records on *C. sativus* published between 2000 and 2021. The global literature output on saffron showed a consistent increase, with the number of publications rising from 39 in 2000 to 413 in 2021, representing an average annual growth rate of approximately 11.05%. The highest number of publications (451 papers) was recorded in 2020. A total of 849 conference papers and original articles were published from 2000 to 2010. This represents only 22.74% of the total number of publications. It is noteworthy that the number of papers increased every year from 2011 to 2021, except for 2012, 2019, and 2021 when the number of publications decreased (Figure 1). Starting from the same period, the overall number of publications has grown intermittently, with over 2000 publications, highlighting a developing focus on the saffron plant. The quantity of research output in this period suggests an intensified concern for *C. sativus*. This may be ascribed to its significance and potential applications in other fields. Many studies have reported that every part of the saffron plant has important benefits [13–16]. This growing consciousness is resulting in expanded uses of saffron in pharmacology, pharmacy, food science and technology, agronomy, microbiology, and horticulture, among other uses.

### Authors' participation (2000-2021)

Based on the WoS data, 13 authors had over 30 published papers on the saffron plant between 2000–2021. These authors collectively published 619 articles, with an average of about 47 articles per author. Three authors published more papers than the above-mentioned average. These authors surpassed the average publication count: H. Hosseinzadeh with 126 articles, G.L. Alonso with 78, and M. Carmona with 55 (Table 1). Considering article quality/impact, the top 13 authors were cited 19,709 times in 619 articles, with an overall average of 30.3 citations per article between 2000 and 2021.

**Table 1:** Most productive authors publishing papers on saffron (2000-2021)

Authors	Number of published papers	Total citations	Average citation per paper	h-index
Hosseinzadeh H	126	5452	43.3	43
Alonso GL	78	2110	27.1	29
Carmona M	55	1739	31.6	25
Gomez-Gomez L	43	1393	32.4	20
Bathaie SZ	41	1402	34.2	20
Fernandez JA	41	597	14.6	15
Ahrazem O	40	919	23.0	19
Rubio-moraga A	34	874	25.7	18
Akhondzadeh S	33	1571	47.6	18
Boskabady MH	33	933	28.3	18
Razavi BM	33	818	24.8	16
Tsimidou MZ	32	605	18.9	15
Tarantilis PA	30	1296	43.2	19
Total	619	19709	30.3	21

**Figure 2:**
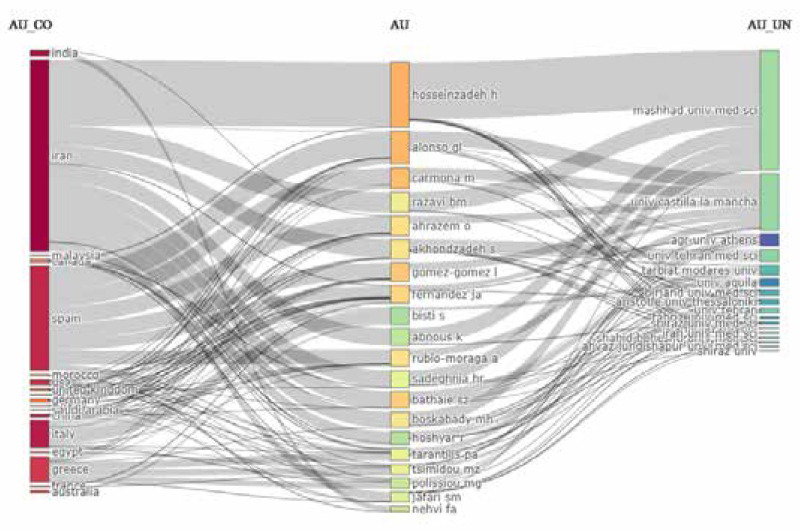
Most 20 productive authors, countries, and universities on *C. sativus* research (2000–2021)

Figure 2 shows a three-field graph for the 20 most productive authors, countries, and universities. Iran, Spain, and Italy were the top-performing countries, while Meshhad Med Science University (Iran) and Castilla La Mancha University (Spain) were the top organizational contributors. The authors H. Hosseinzadeh (mainly with Mashhad Med Science University), G.L. Alonso, and M. Carmona (mainly with Castilla La Mancha University) did not demonstrate a significant collaborative trend for either institutions or countries.

### Collaboration network of countries

**Figure 3:**
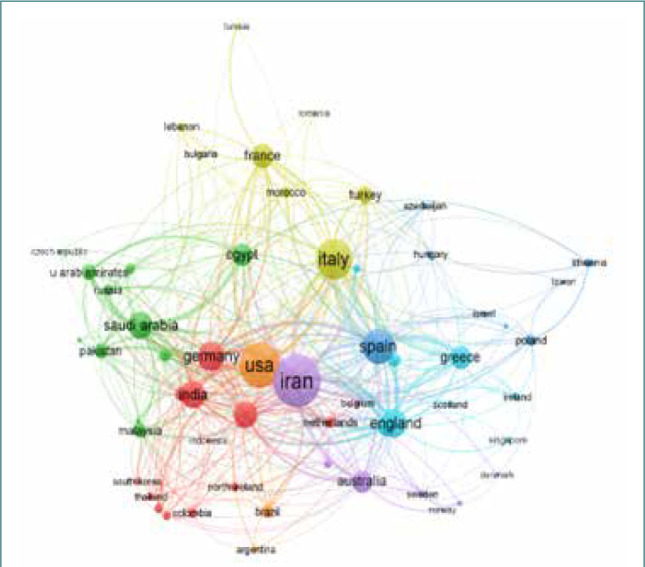
International collaborations network with countries with at least 5 publications (based on the WoS database)

The collaborative networks of the selected countries, with at least 5 published papers, were examined with the VOS viewer. Only fifty-nine countries met the minimum threshold and were aggregated into 7 clusters (Figure 3). The combination of the individual circles results in a cluster, and these are connected with lines to represent the collaborative relationships or networks and their strengths [17,18]. The top-ranked countries are illustrated with larger circles than the low-performing countries. For the 59 outputs, the analysis resulted in a total link strength of 1,176 and 436 links. Iran (264), the United States (210), and Italy (174) had the greatest total link strengths.

Cluster 1 (red) contains 12 countries. Germany and India had the highest collaborative network with other countries in this cluster. Their total link strengths are 103 and 94, respectively. In cluster 2, 11 countries are represented in green. Saudi Arabia and Egypt have the greatest total link strengths of 94 and 67, respectively, while in cluster 3 (9 countries pictured in blue), Spain leads with a link strength of 167. In cluster 4 (8 countries depicted in yellow), Italy has the greatest collaborative network with a total link strength of 174. In cluster 5 (represented in purple), composed of 8 countries, Iran has the strongest collaborative networks. This is expected since the country has the maximum number of publications. In clusters 6 (turquoise) and 7 (orange), England (102) and the USA (210) have the highest total link strength. In summary, the majority of the 59 countries working on saffron research are collaborating. However, there is a continuing need to develop additional collaborative networks among countries with lower rankings and those with higher rankings to better exploit the potential of saffron.

### Journal participation in *C. sativus* research (2000-2021)

Table 2 represents the most important journals in which saffron research articles were published. According to the WoS database, 1,508 journals and proceedings published research studies on *C. sativus* between 2000 and 2021. This section focuses only on journals with a minimum of 30 documents. There were 10 journals that met this threshold, with the Journal of Food Chemistry, at 76 outputs, reporting the highest number of papers on saffron, followed by the Planta Medica Journal, with 55 documents. Others include Molecules (49), Iranian Journal of Basic Medical Sciences (48), Phytotherapy Research (48), Avicenna Journal of Phytomedicine (44), and Journal of Agricultural and Food Chemistry (44).

**Table 2:** Most important journals publishing world papers on saffron (2000-2021)

Journals	Total publications	Total citations	Average citations per journal
Food Chemistry	76	2504	32.9
Planta Medica	55	656	11.9
Molecules	49	735	15.0
Iranian Journal of Basic Medical Sciences	48	1501	31.3
Phytotherapy Research	48	2398	50.0
Avicenna Journal of Phytomedicine	44	761	17.3
Journal of Agricultural and Food Chemistry	44	2139	48.6
Food Science Technology and Nutrition	37	79	2.1
Saffron Science Technology and Health	36	66	1.8
Industrial Crops and Products	32	524	16.4

The number of citations per journal serves as a strong index of influence and research [19]. The average citation per journal of the Journal of Phytotherapy Research was on top (50). This was followed by the Journal of Agricultural and Food Chemistry (48.6) and Food Chemistry (32.9) (Table 2).

### Co-occurrence of keywords related to *C. sativus*

**Figure 4:**
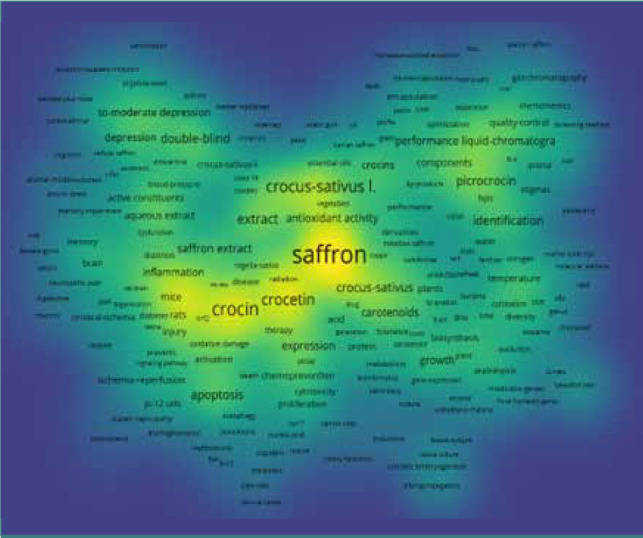
Density map of clusters based on WoS database

**Figure 5:**
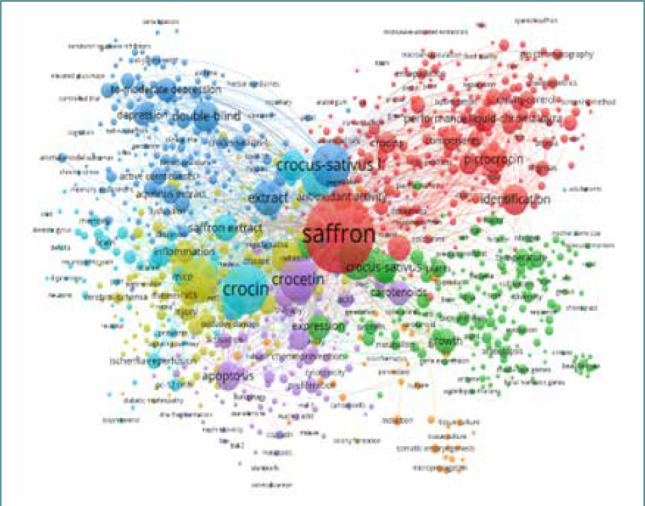
Terms map based on WoS database, made in VOS viewer software

To make the bubble map more efficient, words/terms appearing at least 5 times in the published papers were examined and visualized. Of 13,258 keywords used, 1,067 reached the selected threshold, and 2 were removed manually. The results obtained are displayed in the form of network visualization and density diagrams. According to the terms and density maps (Figures 4 and 5), seven clusters were generated. However, the most significant keywords were saffron, with 1,333 occurrences, followed by crocin, oxidative stress, crocetin, and *Crocus sativus* L., with 563, 410, 408, and 430 occurrences, respectively.

### Saffron production and transformation

**Figure 6:**
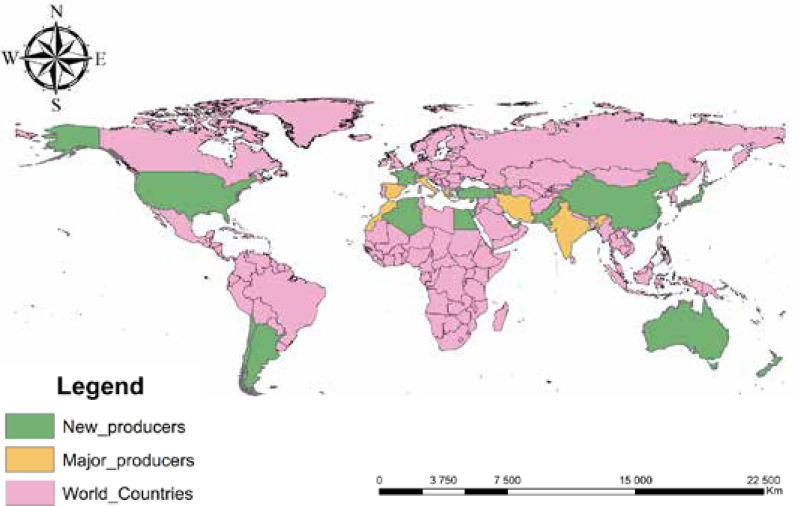
World map with saffron production countries

Saffron is cultivated in different areas and territories across the globe (Figure 6). The main producing countries are the following: Iran (mainly Khorasan, Fars, Kerman, and Yazd), India (Kashmir, Jammu, Uttar, Pradesh, and Himachal Pradesh), Spain (Castile-La Mancha), Italy (Aquila, Cerdeña, San Gimignano, and Emilia-Romagna), Morocco (mainly in the Taznakhte and Taliouine areas), and Greece (Kozani). Recently, there have been new areas of saffron cultivation which include Australia, Azerbaijan, Egypt, France, Israel, Japan, Turkey, Pakistan, United States, New Zealand, China, Argentina, Chili, United Arab Emirates, and Switzerland [1,2,20]. Cultivating saffron in all these areas with different climatic conditions demonstrates its adaptability and undemanding nature.

Saffron is a perennial plant that takes birth from a corm and develops in a wide variety of ecological edaphic and climatic conditions, including altitudes ranging from sea level to 2000m [21]. Saffron corms have a 5-month dormancy period, during which they do not require much water. In fact, saffron cultivation is suitable in arid or semi-arid areas where the water deficit is extreme in summer and temperatures range from –20/–15ºC in winter to 35–45 ºC in summer [2,21].

According to Menia *et al*., saffron grows on a range of soil types but prefers well-drained, loose clay calcareous soils that are friable, allowing for easy root penetration [22]. A recent study published by Pirasteh-Anosheh *et al*. shows that it is possible to cultivate saffron in semi-saline water (2.9 dS m^−1^) and in saline soil (5.8 dS m^−1^). Conventional cultivation of saffron is initiated by ploughing the soil. Deep ditches of about 20 cm or more are created, and saffron corms are placed at the bottom and then covered with soil. Every country has a different planting season, but usually, it takes place in late May to early October. Harvesting is generally carried out from October to November and consists of plucking the flowers and separating the stigmas. In most countries, it is usually performed manually by women [2]. The next step is drying. The quality of the produced stigmas is influenced by this extremely sensitive process [23]. Depending on the country, different drying techniques are used. In Morocco, Iran, and India, the traditional methods of dehydrating stigmas in the sun or the shade for 3–10 days until the humidity content is reduced to about 8–10% are still used. In other countries, drying is carried out employing oak embers, a gas cooker, live vine shoot charcoal, or, to a lesser extent, an electric coil [21]. A recent study by Chen *et al*. showed that freeze drying, infrared drying, and microwave drying were better for preserving the bioactive and aroma compounds of saffron [24].

Currently, global saffron production worldwide is projected at 408 tons. Iran, India, Greece, Afghanistan, Morocco, and Spain are the major producers and exporters. Iran is ranked first with about 90% of world production and 108000 ha of cultivated areas [25].

In addition to the stigma, the most important part of saffron, a large quantity of saffron by-products is produced during stigma processing. In fact, for the production of 1 kg of dried saffron stigma, around 80 kg of *C. sativus* flowers are needed [26]. Unfortunately, such by-products are less valorized and less important economically. They are mainly used as fertilizer or animal feed [27]. Few studies have focused on the other parts of the saffron plant, although the presence of kaempferol and antioxidant activities has been reported by many researchers. Many studies have shown that saffron petals present immense potential for phytopharmaceutical and nutraceutical purposes [28, 29].

### Chemical composition

#### Major compounds

The chemical composition of saffron is well documented. Many previous studies carried out in different countries have reported the proximate composition of *C. sativus* as follows: water (10%), moisture (10%), various sugars (63%), proteins (12–14%), amino acids (12%), minerals (5%), fats (5-8%), starch (6–7%), pectins (6%), gums and dextrins (9–10%), vitamins (B1 (riboflavin), B2 (thiamine) (0.3–1.38%), glucose (7-8%), fructose (1-2%), pentosanes (6-7%), pigments, alkaloids, and essential oils (0.3%) [21,26,30–33]. Minerals, heavy metals, and amino acids reported in saffron stigmas are summarized in Table 3 [34–36].

**Table 3:** Minerals, heavy metals, and amino acids in saffron (*C. sativus*)

Compounds	Content (mg/100 g)	Compounds	Content
Aspartic acid	0.04 – 0.048	Iron	53 – 160 µg/g
Glutamic acid	0.017 – 0.036	Nickel	1.19 – 2.8 µg/g
Threonine	0.019 – 0.026	Potassium	8200 – 12200 µg/g
Serine	0.016 – 0.023	Sodium	25 – 87 µg/g
Alanine	0.13 – 0.17	Zinc	16 – 22.7 µg/g
Proline	0.057 – 0.08	Copper	6 – 10 µg/g
Leucine	0.017 – 0.020	Manganese	13 – 23 µg/g
Valine	0.022 – 0.037	Lithium	27 – 188 ng/g
Isoleucine	0.012 – 0.019	Calcium	218 – 568 µg/g
Glycine	0.007 – 0.016	Magnesium	1130 – 1760 µg/g
Lysine	0.026 – 0.027	Boron	13.1 – 17.1 µg/g
Histidine	0.012 – 0.019	Chromium	0.574 – 1.8 µg/g
Arginine	0.007 – 0.012	Cobalt	54 – 171 ng/g
Tyrosine	0.008 – 0.011	Cadmium	15 – 181 ng/g
Phenylalanine	0.012 – 0.03	Arsenic	11 – 63 ng/g
		Phosphorus	3223 – 4367 ng/g

The three main chemicals found in saffron stigmas are crocin, safranal, and picrocrocin [26]. These three compounds have undergone substantial research, and multiple studies have indicated that they are responsible for the unique properties and several biological activities of saffron [33]. Crocin is produced when crocetin is esterified with various glucosides and is responsible for the golden yellow-orange color of saffron. Safranal (C_10_H_14_O, 2,6,6-trimethyl-1,3-cyclohexadiene-1-carboxaldehyde) and picrocrocin are produced from the degradation of zeaxanthin and belong to the monoterpenoid class [26, 37, 38]. The characteristics of crocin, picrocrocin, and safranal are summarized in Table 4.

**Table 4:** Frequency of liver hydatid cysts according to Gharbi WHO Classification [10]

Compounds	Main characteristics	References
Crocin (C_44_H_64_O_24_) MW: 976.96 g/mol	- Hydrophilic carotenoid is the most abundant in saffron, with about 80% of the total chemical constituents of saffron. - Responsible for the dazzling golden–yellow–red shade of the spice. - Crocin exhibits an antioxidant effect and protects cells and tissues from oxidation (against oxidative stress). - Crocin is stable in hard conditions. - Crocin presents a maximum absorbance at 440nm. - Crocin is water soluble and is used in many industries as a coloring agent. - Total crocin amount might vary from 6 to 16% of the dry saffron mass.	[2] [26, 39]
Safranal (C_10_H_14_O) MW: 150. 21 g/mol	- Constitutes the main constituent of the essential oil of saffron, about 30 to 70%. - Responsible for the specific aroma of saffron. - It is a monoterpene aldehyde aglycon of picrocrocin formed during dehydration and storage after harvest because of the action of β-glucosidase.\ - The λ_max_ for safranal is at 330 nm. - It is made from 0.001 to 0.006% of saffron dry matter. - Safranal amount may range from 0.04 to 0.48%.	[40–44]
Picrocrocin (C_16_H_26_O_7_) MW: 330.37 g/mol	- Responsible for the specific flavor and bitter taste of saffron. - Makes approximately 1 to 13% of the dry matter of saffron. - Picrocrocin-a precursor of safranal is a monoterpene glycoside found in the essential oil of saffron. It is the second most abundant compound in the essential oil of saffron. - Presents a maximum absorbance at λ=254 nm.	[41, 44, 45]

#### Secondary metabolites

Recent studies have reported that the saffron stigma is an excellent reserve of bioactive compounds, with more than 150 compounds identified [2, 9, 32]. Most of these compounds belong to different classes, namely, carotenoids, flavonoids, monoterpenes, and monocyclic aromatic hydrocarbons.

Carotenoids identified in saffron are as follows: crocin, crocetin (8,8' -Diapo-ψ,ψ-carotenedioic acid), dimethyl-crocetin, methyl-crocetin, crocetin-di-(β-D-glucosyl)-ester, 13Z-crocin, Zeaxanthin, crocetin-di- (β-neapolitanosyl)-ester, crocetin-1-all-O-β-gentiobiosyl-ester, lycopene (ψ,ψ-carotene), cis-crocetin- (β-gentiobiosyl)-(β-neapolitanosyl)-ester, cis-crocetin (β-D-triglucoside)-(β-D-gentibiosyl) ester, crocetin- (β-gentiobiosyl)- (β-neapolitanosyl)-ester, α-carotene, crocetin-mono-(β-D-glucosyl)-ester, crocetin (β-D-neapolitanose)-(β-D-glucosyl) ester, β-carotene (β, β-carotene), cis-crocetin (β-D-neapolitanose)-(β-D-glucosyl) ester, crocetin-mono-(β-D-gentiobiosyl)-ester, crocetin (β-D-triglucoside)-(β-D-gentibiosyl) ester, γ-carotene (β,ψ-carotene), crocetin-(β-D-gentiobiosyl)-(β-D-glucosyl)-ester, Phytoene, Lycopersene, ζ-carotene (Tetrahydrolycopene), and Phytofluene [6, 9, 40, 46–50].

Many compounds belonging to the flavonoids family were identified in saffron as follows: Kaempferol, Kaempferide, Astragalin, kaempferol-3-O-sophoroside-7-O-glucoside, kaempferol-3,7,4' -triglucoside, kaempferol-7-O-sophoroside, sophoraflavonoloside, kaempferol-3-O-β-D-(2-O-β-D-6-O-acetylglucosyl)-glucopyranoside, kaempferol 3-O-β-D-(6-O-acetyl)-glucopyranoside, kaempferol 7-O-β-D-glucopyranoside, kaempferol 3,7-di-O-β-D-glucopyranoside, kaempferol-3-O-β-D-(6-O-acetyl)glucopyranoside-7-O-β-Dglucopyranoside, kaempferol-3-O-β-D-(2-O-β-D-6-acetylglucosyl)glucopyranoside-7-O-β-D-glucopyranoside, isorhamnetin-4' -O-α-L-rhamnopyranosyl(1 → 2)-β-Dglucopyranoside (crosatoside A), Helichrysoside, isorhamnetin-3,4' -diglucoside, isorhamnetin-3-O-robinobioside, isorhamnetin-3-O-glucoside, Rutin, Apigenin, vitexin, isoorientin, naringenin, orientin, populin, myricetin, quercetin, and rhamnetin [6, 9, 26, 50–55].

Numerous studies have reported many monoterpenes isolated from saffron including: (4R)-4-hydroxy-2,6,6-trimethylcyclohex-1- enecarbaldehyde 4-O -[βDglucopyranosyl (1 → 3)-β-Dglucopyranoside], (4S)-4-(Hydroxymethyl)-3,5,5-trimethylcyclohex-2-enone-β-Dglucopyranoside, (4R)-4-hydroxy-2,6,6-trimethylcyclohex-1-enecarbaldehyde O-β-D- gentiobioside, (4R)-4-hydroxy-2,6,6-trimethylcyclohex-1-enecarboxylic acid O-β-D-glucopyranoside, 6-hydroxy-3-(hydroxymethyl)-2,4,4-trimethylcyclohexa-2,5-dienone 6-O-β-D-glucopyranoside, (5S)-5-hydroxy-7,7-dimethyl-4,5,6,7-tetrahydro-3Hisobenzofuran-1-one O-β-Dglucopyranoside, (1R,5S,6R)-5-(hydroxymethyl)-4,4,6-trimethyl-7-oxabicyclo[4.1.0] heptan-2-one O-β-Dglucopyranoside, 5-hydroxy-7,7-dimethyl-4,5,6,7-tetrahydro-3Hisobenzo-Furanone 5-O-β-D-gentibioside, 4-hydroxymethyl-3,5,5-trimethylcyclohex-2-en-1-one 4-O-β-D-gentibioside, (4R)-4-hydroxy-3,5,5-trimethylcyclohex-2-enone 4-O-β-Dglucopyranoside, (4S)-4-hydroxy-3,5,5-trimethylcyclohex-2-enone 4-O-β-Dglucopyranoside, (2Z)-3-methylpent-2-enedioic acid 1-[1-(2,4,4-trimethyl-3,6-dioxocyclohexenyloxy)-O-β-Dglucopyranosid-6-yl] ester, (1R)- 3,5,5-trimethylcyclohex-3-enol O-β-D-glucopyranoside, (4S,3' R)-4-Hydroxy-4-(3' -hydroxy-1' -butenyl)-3,5,5-trimethyl-2- cyclohexen-1-one 3' -O-β-Dglucopyranoside, safranal (2,6,6-trimethyl-1,3-cyclohexadiene-1-carboxaldehyde), 2,6,6-trimethyl-1,4-cyclohexadiene-1-carboxaldehyde, 2,6,6-trimethyl-4-hydroxy-1- cyclohexen-1-carboxaldehyde, 2,4,4-trimethyl-3-formyl-6-hydroxy-2,5-cylohexadien-1-one, 4-hydroxymethyl-3,5,5-trimethylcyclohex-3-enol, crocusatin-B, crocusatin-C, crocusatin-D, crocusatin-F, crocusatin-G, crocusatin-H, crocusatin-I, crocusatin-J, crocusatin-K, crocusatin-L, 3-hydroxy-β-ionone, isophorone, 3,5,5-trimethyl-4-hydroxy-1-cyclohexanon-2-ene, 3,5,5-trimethyl-1,4-cyclohexadione, 3,5,5-trimethyl-1,4-cyclohexadion-2-ene, 3,5,5-trimethyl-2-hydroxy-1,4-cyclohexadion-2-ene, crocusatin-A, crocusatin-E [6, 9, 40, 50, 53, 56–58].

Monocyclic aromatic hydrocarbons found in saffron are sodium (2S)-(O-hydroxyphenyl) lactate, methylparaben, protocatechuic acid methyl ester, protocatechuic acid, 4-hydroxybenzoic acid, 4-hydroxyphenethyl alcohol, benzoic acid, 1-O-(4-hydroxybenzoyl)-β -D-glucose, p-coumaric acid, vanillic acid, methylvanillate, 3-hydroxy-4-methoxybenzoic acid, β-(p-hydroxyphenyl) ethanol-α-O-α-Lrhamnopyranosyl(1 → 2)-β Dglucopyranoside(crosatoside B), 2,4-dihydroxy-6-methoxyacetophenone-2-β-D-glucopyranoside, 2,3,4-trihydroxy-6-methoxyacetopenone-3-β-D-glucopyranoside, benzyl O-β-D-glucopyranoside, 2-phenylethyl O-β-D-glucopyranoside [9, 52, 53, 57, 59]. Several nitrogen-containing compounds were also identified in saffron namely 5-methyluracil, pyridin-3-ylmethanol, uracil, nicotinamide, adenosine, harman, tribulusterine, and 1-(9H-β-carbolin-1-yl)-3,4,5- trihydroxypentan-1-one [9, 52, 53].

### Saffron quality control and authenticity

The quality of saffron is related to its low water content and high content of particular scents (safranal) and specific coloring (crocin and picrocrocin). The International standards ISO 3632-1 and ISO 3632-2 2011 have been developed to monitor the quality of marketed saffron.

Also known as red gold, the high price of saffron is due to many factors, including its large number of applications, increased demand in the market, limited areas of production, and for the most part, manual means of production and low yield. Due to its high market value, saffron is one of the most targeted products for fraudulent practices. Disparate methods are employed by cheaters to maximize profit and delude consumers by adding foreign materials (biological, artificial, or synthetic adulterants) that have a similar appearance to *C. sativus*. Adding similar plants such as *Calendula officinalis* L., *Buddleja officinalis* Maxim., *Gardenia jasminoides* Ellis., *Curcuma longa* L., and *Carthamus tinctorius* L, and mixing with low-quality saffron or adding various parts of the saffron plant such as petals and stamens or even other species of crocus like *Crocus vernus* and *Crocus speciosus*, are common fraudulent practices [91-94,100]. Incorporating chemicals such as potassium nitrate, starch, potassium hydroxide, sodium, lactose, monopotassium tartrate, glucose, barium sulphate, sodium borate, and calcium carbonate is another common illegal behavior. Powdered saffron is usually adulterated by adding various synthetic dye and chemicals like allura red, magenta III (new fuchsin), azorubine, rocelline, carminic acid, cochineal A red, tartrazine, naphthol yellow, sudan II, sunset yellow, picric acid, quinoline, rhodamine B, emaranth, ponceau 4R and red 2G, martins yellow, tropeolina, erythrosine, and fucsina [62-64,73,100].

In addition to the aforementioned methods of adulteration, there are several unusual practices employed to increase the weight of saffron. These include soaking saffron stigmas in syrups, honey, glycerin, or olive oil and adding animal tissue such as dried meat fibers [100].

Researchers have made significant efforts to combat these fraudulent practices. Numerous techniques have been developed to detect adulterants, as outlined in Table 5. These techniques encompass various approaches, including physical, chromatographic, molecular, DNA-based, sensor-based, and spectroscopic strategies [101]. Physical procedures are the oldest, most fundamental, and most straightforward ways to obtain physicochemical information regarding the purity of saffron, including its ash content, morphological properties (size, shape, appearance, etc.), color, and flavor. One of the physical ways for saffron adulteration detection is using microscopic and macroscopic equipment to identify impurities in saffron. The main advantages of physical procedures are their simplicity, ease of use, and lack of sample preparation requirements. However, these methods have certain limitations, including the inability to quantify adulterants and a lack of sensitivity, precision, specificity, and reproducibility. Chromatographic techniques are widely employed for saffron authentication and are considered among the most commonly used methods. Several chromatographic tools are used, including HPLC (High-Performance Liquid Chromatography), GC (Gas Chromatography), and TLC (Thin Layer Chromatography). In addition to the standalone chromatographic techniques mentioned earlier, various combinations of these techniques with other techniques and detectors are utilized in saffron authentication. These include HPLC-DAD, LC-MS/MS, HPLC/PDA/MS, UHPLC-MS/MS, GC/MS/MS, and HS-GC-FID [62–67]. These combinations offer several advantages in detecting a wide range of adulterants, including biological, synthetic, and chemical adulterants, both volatile and non-volatile compounds.

**Table 5:** Techniques used to detect adulteration in saffron

Methods category	Technique used	Description	Targeted compounds or adulterants	References
Physical methods	Coloration Ashing Microscopic analysis	The best-known method is to immerse the saffron in a water solution and see the color released; the pure saffron releases yellow-orange color slowly, giving the aroma of saffron. Diphenylamine (DPA) is used to check the purity of saffron; after adding saffron, the solution becomes blue and then dark reddish brown. Ash content is used to check inorganic contaminations; ash should be inferior to eight percent. Cell types and tissue structure could also be used to check the purity of saffron using microscopic analysis.	Biological adulterants Inorganic salts Chemical adulterants	[60,61]
Chromatographic and electrophoresis techniques	Thin Layer Chromatography (TLC) High-performance liquid chromatography (HPLC) Gas chromatography Electrophoresis	TLC technique detects different adulterants in saffron, especially artificial colorants. Layer chromatography combined with other techniques such as image analysis (TLC-IA), Raman spectroscopy, and chemometrics, high-performance thin-layer chromatography (HPTLC) coupled with multivariate image analysis (MIA) were proposed as reliable and fast techniques to check the authenticity of saffron. HPLC-DAD UHPLC-DAD-MS method for detecting adulteration of saffron using gardenia. HPLC in conjunction with PDA-ESI-MS (Photodiode-Array-Electrospray Ionization- Mass Spectrometry) Ultra-High-Performance Liquid Chromatography- High-Resolution Tandem Mass Spectrometry) LC-MS (liquid chromatography-mass spectrometry) Liquid Chromatography-Quadrupole-Time of flight-Mass Spectrometry LC-QTOF-MS Gas chromatography-mass spectrometry GC-MS. Gas chromatography-combustion- isotopic ratio mass spectrometry GC-C-IRMS combined with chemometrics. Headspace Gas-Chromatography with Flame Ionization Detector in conjunction with multivariate data analysis techniques HS-GC-FID. Solid-phase microextraction gas chromatography-mass spectrometric, SPME-GC-MS. 1D-Gel Electrophoresis for proteins determination	Artificial colorants. Amino-acids. Natural adulterants (safflower, marigold, and turmeric) Metabolic fingerprints Gardenia kaempferol derivatives Safranal and 2-caren-10-al No targeted compounds Volatile profile. Plant adulterants	
Spectroscopic techniques	UV–Vis Spectroscopy Fourier Transform Infrared Spectroscopy (FTIR) Nuclear Magnetic Resonance (NMR) Mass spectrometry (MS) Raman Spectroscopy Near-infrared (NIR) spectroscopy Fluorescence	UV–Vis spectrometry UV–Vis spectrophotometry combined with linear discriminant analysis (LDA) Transmittance Fourier Transform Infrared (FT-IR) Spectra Attenuated Total Reflectance Fourier Transform Infrared (ATR)-FTIR Diffuse Reflectance Infrared Fourier-transform spectroscopy (DRIFTS) combined with PLSDA and PLS. FT-IR spectroscopy, in combination with other techniques, colorimetry, UV–Vis spectrometry, Reverse Phase-High Performance Liquid Chromatography-Photodiode Array Detector (RP-HPLC-DAD) 1H NMR technique H NMR combined with modelling techniques (OPLS-DA, PLS-DA, and PCA). Matrix-assisted laser desorption/ ionization - mass spectrometry MALDI-MS/MS UHPLC-ESI/QTOF mass spectrometry Inductively coupled plasma mass spectrometry (ICPMS) Isotope-ratio mass spectrometry (IRMS) Direct analysis in real time-high resolution mass spectrometry (DART-HRMS) Proton transfer-mass spectrometry (PTR-MS) Raman spectroscopy combined with chemo-metrics Thin-layer chromatography (TLC) combined with Raman spectroscopy Near-infrared spectroscopy combined with multivariate data analysis Spectrofluorometer	Synthetic red colorants According to ISO Normative 3632 Various artificial food colorants Crocin content Plant-based adulterants Carminic acid Crocetin and picrocrocin Plant-based adulterants Gardenia, turmeric (Other parts of the flower) Mineral adulterants, Volatile compounds Plant-based adulterants Plant-based adulterants Synthetic dyes	[73, 74] [75 - 79] [80] [81, 82] [83 - 85] [86, 87] [77] [63] [88]
Molecular techniques		Loop-mediated isothermal amplification (LAMP) PCR technique Bar-HRM DNA-based plastid genes matK High-resolution melt analysis Conjoined with universal DNA barcoding regions (Bar-HRM), DNA barcodes Recombinase polymerase amplification in conjunction with lateral flow immunoassay strip (RPA-LFD)	Plant-based adulterants	[89- 94]
Sensor-based technique		Electronic nose (E-nose), voltammetric electronic tongue (VE-tongue), E-nose based on metal oxide sensors (MOX), E-tongue,	Volatile compounds, safflower, pale yellow style of Saffron, corn fibres dyed with extract of beetroot, dyed corn silk, Tartrazine, and Methyl orange	[95 - 99]
Elemental analysis	Elemental analyzer	C–H–N elemental analyzer	Bio-element stable isotope	[100]

Nevertheless, they are time-consuming, require sample preparation, have higher costs, and require highly trained personnel. The most popular spectroscopic techniques used in saffron authentication are UV–VIS spectroscopy, Raman spectroscopy, fluorescence spectroscopy, near-infrared spectroscopy (NIR), laser-induced breakdown spectroscopy (LIBS), Fourier-transform infrared spectroscopy (FTIR), mid-infrared spectroscopy (MIR), ion mobility spectrometry (IMS), nuclear magnetic resonance spectroscopy (NMR). Molecular and DNA-based approaches have recently gained popularity in saffron adulteration detection due to their relatively low cost. These approaches include techniques such as polymerase chain reaction (PCR), arrays (DNA array and protein array), hybridization, and sequencing techniques [91-98]. Sensor-based techniques and elemental analysis are also employed in saffron adulteration detection [95–100].

In addition to the techniques already in use, several emerging procedures have significant potential in detecting food adulteration and could be used in the future to authenticate and detect saffron adulteration. Among others, hyperspectral imaging (HSI), electron spin resonance (ESR) spectroscopy, and terahertz spectroscopy are worth mentioning [100-102]. Alighaleh *et al*. proposed a new approach based on deep neural networks and RGB photos taken under uncontrolled/unstructured conditions to detect genuine saffron [103]. In another work, a simple, non-chemical, non-destructive, fast, and accessible method using visible-near infrared hyperspectral imaging (Vis-NIR-HSI) combined with a new chemometric strategy based on mean-filed independent component analysis (MF-ICA) and multivariate classification techniques is proposed to detect fake saffron [104].

### Saffron uses

#### Therapeutic uses

Due to its specific composition, several studies of saffron have reported a broad spectrum of biological activities. These include: antioxidant, anti-tumor, antianxiety, antiviral, anti-inflammatory, antigenotoxic, anticancer, anti-atherosclerotic, anti-diabetic, hypotensive, hypoglycemic, antihyperlipidemic, antidegenerative, anti-tumor, insulin resistant reducers, anti-convulsant activity, anti-nociceptive, anti-Alzheimer, aphrodisiac, stimulant, anti-poison, livotonic, lactogogue, anticancer, nervine tonic, cardiac tonic, carminative, immune stimulator, diaphoretic, diuretic, sedative, emmenagogue, relaxant, febrifuge, anti-stress, antiulcer, antimutagenic, antigenotoxic, memory, and learning enhancer, anticonvulsant, antidepressant, blood pressure regulator, oxygen boosting of tissues, and bronchodilator [105–116]. These properties are basically due to apocarotenoids, which are bioactive chemicals and include crocin, crocetin, and safranal. Some studies have indicated that saffron can prevent Alzheimer's disease [39], heart disease [117], mild-to-moderate depression [118, 119], cardiovascular diseases [120], gastrointestinal disorders, severe headaches [121], neurodegenerative and psychiatric disorders [122,123], premenstrual syndrome, and anxiety [116]. It is also considered effective in improving memory and learning skills [25, 124].

Saffron is used in folk medicine and the Ayurvedic health system as a sedative, expectorant, anti-asthma, emmenagogue, and adaptogenic agent [125]. Additional virtues ascribed to saffron are around the gastro-intestinal and genital system, in particular, the ability to stimulate the stomach, decrease appetite, cure hemorrhoids, prolapse of the anus, restrain intestinal fermentations, and help in the treatment of amenorrhea [126]. Saffron has been recommended for use in the reduction of pain and inflammation. It has also been proposed as an analgesic agent [127]. Moreover, saffron extracts have been used to treat fever, wounds, back pain, abscesses, gingivitis, and pain related to the eruption of first teeth in children [121]. It is often used to reduce blood cholesterol levels and thus the severity of atherosclerosis, resulting in a reduced risk of myocardial infarction [128]. A study by Mashmoul *et al*. proved the effectiveness of a cream using an extract of saffron in treating burns induced by heat [129]. Finally, Khazdair *et al*. [130] showed that saffron effectively improves sleep quality.

#### Cosmetology and perfumery uses

In addition to therapeutic uses, saffron is used for cosmetics and perfumery [2]. It is reported to have an anti-sun effect, so it can be used as a natural UV absorber to protect the skin from harmful rays [7, 131]. As mentioned in therapeutic uses, saffron has anti-carcinogenic properties and can prevent skin cancers and depigmentation. Moreover, saffron has been suggested to exhibit an anti-rhythmic effect on human skin by reducing melanin. Saffron has long been used in cosmetics as a natural pigment, very effective in lightening the skin [132–134]. Clinical trials on the anti-pruritic effects of saffron and its considerable overall positive effect on the skin were observed [135]. Mzabri *et al*. [7] discussed saffron’s anti-aging effects, its use in treating blemishes such as acne, and its aptitude in exfoliating and improving blood circulation for facial skin.

#### Culinary uses

Culinary purposes are one of the main uses of saffron. Worldwide, most of the saffron is used in cooking for flavoring and as a dye. Its unique flavor is described by chefs and saffron specialists [2]. Its three main compounds, crocin, picrocrocin, and safranal, determine the intensity of the color, the power of the flavor, and the strength of the aroma, respectively. Saffron is used to color a large variety of dishes and drinks in many countries. In India, it is used mainly in some dishes such as “Kheer,” “Biryani,” “Kashmiri pulao,” and "Kehwa." Italy uses it in “Risotto alla Milanese,” France in “Bouillabaisse,” and Spain in “Paella Valenciana” [22]. It is also a key ingredient in a variety of pastries and bread, many of which celebrate a secular or religious holiday, such as the Gugelhupt cake in Europe, St. Lucia buns in Sweden, buns and bread in Cornwall (England), Christmas bread in Estonia, sweets on the Greek islands, and rice pudding during Iranian Shi'ite as well as Jewish holidays [21]. In Morocco, saffron is used to aromatize tea instead of mint and also as a spice in the preparation of various traditional dishes such as “koftas” (meatballs and tomatoes) and “mrouzia” (a sweet and salty dish made with mutton and dill). Saffron is also a central ingredient in the herbal mixture known as chermoula, which flavors many Moroccan dishes [136].

#### Other uses

In addition to the above-mentioned applications, several studies have reported many other potential uses of saffron. Khoulati *et al*. reported that saffron extract could be a bio-stimulant source for some plants, such as tomatoes [137]. Saffron is also used as a dye in the textile industry, though this use has practically disappeared with the development of artificial coloring [138].

## DISCUSSION

In recent years, there has been a significant increase in scientific interest surrounding the saffron plant. This is evident from the growing number of research papers published on saffron-related topics over the past two decades. Analysis of the data from the WoS database reveals a substantial rise in the total number of saffron publications, exceeding 2000 papers. This may be attributed to its significance and potential applications in many fields. Regarding authors' contribution and collaboration network of countries, thirteen authors were found to be the most productive, and six authors exceeded the average impact (30.3), with S. Akhondzadeh (47.6 citations), H. Hosseinzadeh (43.3 citations), and P.A. Tarantilis (43.2 citations) S.Z Bathaie. (34.2 citations), L. Gomez-Gomez (32.4 citations), and M. Carmona (31.6 citations). When comparing these authors using the h-index, three reached a higher level than the average (21) for the same period. As illustrated in Table 1, these authors are H. Hosseinzadeh (43), G.L. Alonso (29), and M. Carmona (25). Iran, Spain, and Italy were the high-performing nations, while Meshhad Med Science and Castilla La Mancha were the top contributor universities. Despite the good production achieved so far, it would be advantageous if these researchers collaborate and connect with international researchers in the future. This would enhance their production and citation.

The saffron industry has witnessed an expansion in the number of producing countries in recent years. This can be attributed to the relatively low input requirements of the saffron plant, making it feasible for cultivation in various regions. Approximately 408 tons of saffron stigma are produced annually worldwide, with Iran holding the leading position as the largest producer. During the stigma processing, a significant amount of saffron by-products are generated. In addition to the stigma, these by-products have also attracted the attention of researchers, and many studies have been carried out on them, showing an immense potential to be used for phytopharmaceutical and nutraceutical purposes [28, 29].

The composition of *C. sativus* stigma is well documented [9, 32]. Crocin, safranal, and picrocrocin are the three principal compounds present in saffron stigmas. The secondary metabolites composition of *C. Sativus* has also been explored, and more than 150 compounds with important biological activities have been isolated. It should be mentioned that the chemical composition of saffron is influenced by many factors, such as soil composition, climatic conditions, location, agronomic practices, harvest time, method processing, and conservation [37,38].

Certainly, the quality of saffron is influenced by its composition, especially water content and particular compounds like safranal, crocin, and picrocrocin. Because of its high price, saffron is known to be one of the most targeted products for different types of adulterations. Tricksters add foreign substances (biological, artificial, or synthetic adulterants) that have a similar aspect to *C. sativus* in order to maximize profit and mislead consumers. To suppress this phenomenon, an international standard, ISO 3632 (ISO 3632-1; ISO 3636-2), has been developed to evaluate the quality of marketed saffron using spectrophotometric and chromatographic measurements. In addition to the international standard ISO, and because of the emergence of pollutants that are difficult to detect and the development of falsification techniques, researchers have proposed several other methods involving chromatographic, molecular, DNA-based, sensor-based, and spectroscopic techniques. More recently, some emerging approaches involving new techniques include hyperspectral imaging (HSI), electron spin resonance (ESR) spectroscopy, terahertz spectroscopy, deep neural network, and RGB photos [91-98, 95-102]. More deep research is needed to explore the effectiveness of such techniques.

The potential uses of saffron have attracted significant scientific research interest. Studies have explored a wide range of applications for saffron, highlighting its diverse biological potential. With a rich history spanning over 3000 years in traditional medicine, saffron has been revered as a panacea in numerous cultures, offering treatment for an extensive range of approximately 90 illnesses [32,108,109]. This demonstrates the excellent pharmaceutical nature of *C. sativus*. Numerous studies have highlighted its potential efficacy in addressing different types of cancers [108, 110], including breast and lung cancer [111], cervical cancer [112], and colorectal cancer [113].

Additionally, saffron has been traditionally used to alleviate symptoms of asthma, cough, flatulence, stomachic disorders, colic, insomnia, chronic uterine hemorrhage, amenorrhea, smallpox, bronchospasm, sexual dysfunction, and infertility [7, 32, 114–116]. Beyond its medicinal use, saffron is used in several cosmetic and perfumery products to care for the skin [7, 131–134]. Culinary uses are one of the most important applications of saffron stigma, used in cooking for flavoring and as a dye in many countries. Based on the available information, the therapeutic uses of saffron are still being explored, and further research is needed to determine its specific applications and effectiveness in treating various conditions. There is a growing trend in the cosmetology and perfumery industries to incorporate natural and healthy elements into their products.

## CONCLUSION

Saffron is the most expensive spice in the world, rich in bioactive compounds, and useful in many fields. Although many studies have been conducted on this plant, some gaps exist. There is an ongoing need to develop collaborative networks in research between countries with lower rankings and those with higher rankings to explore better and utilize the potential of saffron. Expanding the cultivation of saffron to more nations is a viable option since the plant is adaptable to a wide range of pedoclimatic conditions. This expansion can help meet the growing market demand for *C. sativus*.

Furthermore, research on the mechanization of saffron production and processing is essential. Implementing mechanized techniques would improve efficiency, increase availability, and reduce costs associated with saffron production. Introducing other types of drying, such as freeze-drying, would also help obtain a consistently high-quality product. Studies exploring additional saffron by-products could valorize them while increasing the amount farmers earn. Developing more practical, inexpensive, and responsive approaches to detect adulterants that can be routinely used on an industrial scale would greatly enhance consumer confidence, augment interest, and increase the marketability of premium saffron.
